# Successful treatment of isolated bile leakage after hepatectomy combination therapy with percutaneous transhepatic portal embolization and bile duct ablation with ethanol: a case report

**DOI:** 10.1186/s40792-018-0463-y

**Published:** 2018-06-19

**Authors:** Norio Kubo, Norifumi Harimoto, Kei Shibuya, Norihiro Ishii, Mariko Tsukagoshi, Takamichi Igarashi, Akira Watanabe, Kenichiro Araki, Masaya Miyazaki, Hiroyuki Kuwano, Ken Shirabe

**Affiliations:** 1 Department of Hepatobiliary and Pancreatic Surgery, Gunma University, Graduate School of Medicine, 3-39-22 Showa-machi, Maebashi, Gunma 371-8511 Japan; 2 Department of Diagnostic and interventional Radiology, Gunma University Hospital, 3-39-15 Showa-machi, Maebashi, Gunma 371-8511 Japan; 3 Department of General Surgical Science, Gunma University, Graduate School of Medicine, Maebashi, Japan

**Keywords:** Bile leakage, Divided bile duct, Isolated bile duct, Segmental bile leakage, Percutaneous transhepatic portal embolization, Bile duct ablation

## Abstract

**Background:**

Bile leakage after hepatectomy still causes relatively serious problems, and some types of bile leakage are intractable.

**Case presentation:**

We report a case of postoperative isolated bile duct leakage managed successfully by combination therapy of percutaneous transhepatic portal vein embolization (PTPE) and bile duct ablation with ethanol. A 61-year-old man diagnosed with hepatocellular carcinoma underwent partial hepatectomy. On postoperative day 1, bile leakage was detected at the drainage tube. Simple drainage treatment did not improve the situation. He was diagnosed with isolated bile leakage based on fistulogram from the drainage tube that showed the bile duct at segments V and VIII but not the common bile duct. A volume of drainage fluid of 200 mL/day was observed. Combination therapy with PTPE and bile duct ablation with ethanol was planned. After the percutaneous transhepatic cholangiography, the drainage tube was inserted into the bile duct, and PTPE was performed to segments V and VIII. The amount of drainage fluid decreased, and bile duct ablation with ethanol was performed to the isolated bile duct. No complication was found following combination therapy.

**Conclusion:**

In this case, we successfully treated a patient with isolated bile leakage by combination therapy with PTPE and bile duct ablation.

## Background

Recently, it has been possible to perform hepatectomy safely because of improvement of surgical techniques and perioperative management [1, 2]. Recent studies reported that perioperative mortality rates were 2.6 to 3% after major hepatectomy [3, 4]. However, bile leakage has still a relatively high incidence in the range of 4.8–8.7% [1, 2, 5]. Bile leakage may lead to intraperitoneal septic complications, liver failure, and ultimately death. The risk factors for bile leakage have been already described previously [6]. Reported factors that were correlated with the occurrence of bile leakage were male sex, advanced age, tumor size, repeated hepatectomy, preoperative chemoembolization, large liver cut surface, exposure of Glisson’s sheath, duration of vascular occlusion, prolonged operation time, and red cell transfusion [6–9]. Bile leakage after hepatectomy was defined according to the international study group of liver surgery definition, i.e., drainage of fluid with a bilirubin level of three times greater than the serum level after postoperative day (POD) 3 or the need for interventions as the result of bile collections or biliary peritonitis [10]. Most bile leakages are treated with simple drainage. However, some types of bile leakage require interventions such as endoscopic bile drainage or percutaneous abdominal drainage. Furthermore, intractable bile leakage as represented by isolated bile leakage was not improved by drainage alone.

In this report, we present a patient with isolated bile duct leakage after hepatectomy who was successfully treated by combination therapy with percutaneous transhepatic portal embolization and bile duct ablation with ethanol without laparotomy.

## Case presentation

**Fig. 1:**
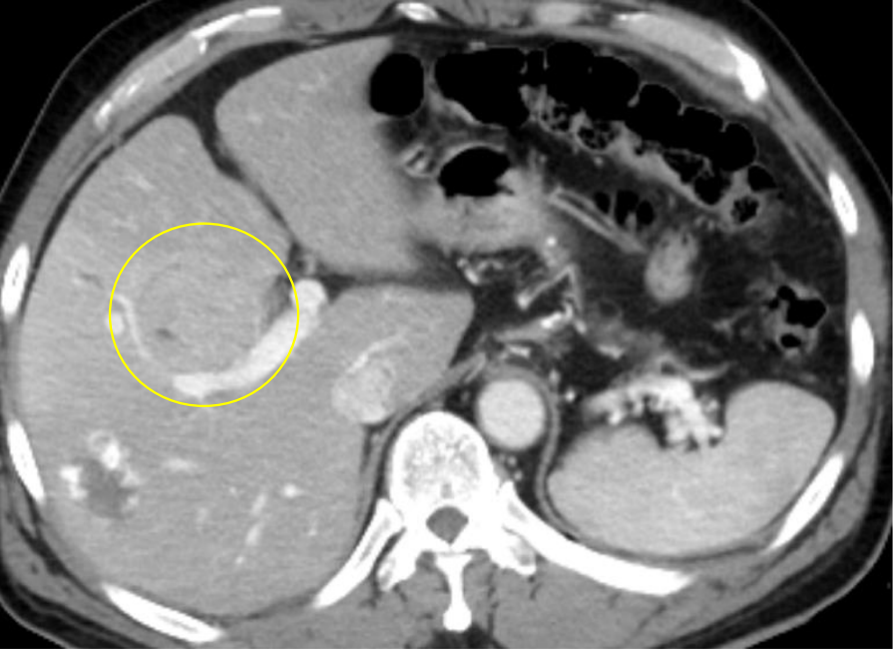
Preoperative abdominal computed tomography showed a 50 × 48 mm hepatic mass that pressed the anterior Glisson

**Fig. 2:**
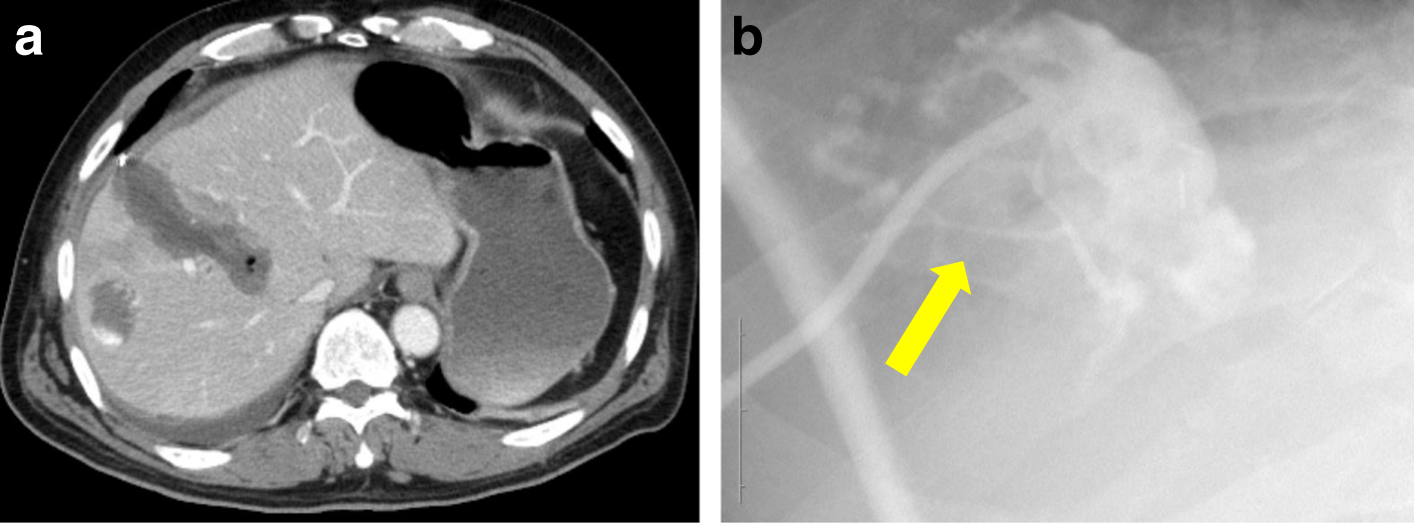
On postoperative day 19, abdominal computed tomography showed the fluid at the liver cut surface (**a**). Cholangiography via the c-tube did not detect the anterior branch of the bile duct (arrows) and does not communicate with the abdominal cavity (**b**)

**Fig. 3:**
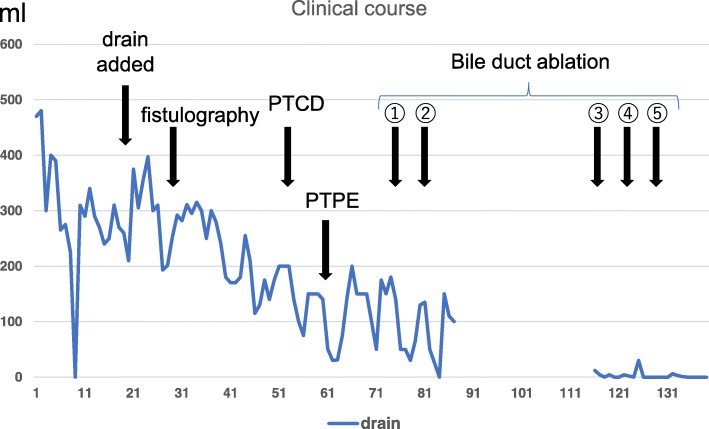
Clinical course of bile duct leakage and drainage amount. The number shows the times of bile duct ablation. PTCD: percutaneous transhepatic cholangiography drainage. PTPE: percutaneous transhepatic portal embolization

A 61-year-old man underwent resection of a part of his tongue due to tongue cancer and was admitted to our hospital for hepatocellular carcinoma with about 5 cm diameter of tumor at the liver segment IV. Computed tomography (CT) showed that the tumor was enhanced during the arterial phase and washed out during the portal phase, and the tumor pressed the right anterior branch to the main branch of Glisson and the middle hepatic vein; hence, the diagnosis of hepatocellular carcinoma was made (Fig. 1). The indocyanine green retention rate at 15 min was 21.8%. Child-Pugh score was A. The patient tested negative for hepatitis B surface antigen and hepatitis C virus antibody. He had a history of excessive consumption of alcohol, and alcoholic liver damage was considered as a possibility. Partial hepatectomy with segments IV + V and cholecystectomy with cystic duct-tube drainage were performed. Intraoperative findings indicated that the anterior branch of bile duct was exposed at the resected area, and some small bile ducts were ligated. On POD 1 following hepatectomy, bile leakage developed from the drain placed in the foramen of Winslow. CT showed fluid collection in the cavity between the liver and fistula to drain. Cholangiography via the endoscopic bile duct enhancement showed no communication between the common bile duct and abdominal cavity. Drip infusion cholangiography (DIC)-CT revealed the bile duct of the peripheral side. We maintained simple drainage to reduce the cavity. On POD 19, this patient had fever and CT revealed that the fluid collection has increased (Fig. 2a). Percutaneous drainage to the cavity near the liver cut surface was performed. Cholangiography via the c-tube did not show the anterior branch of the bile duct. Fistulogram from the drainage tube at the abdominal cavity showed the bile duct at segments V and VIII (Fig. 2b). We diagnosed the bile leakage from the isolated bile duct of segments V and VIII. Further management was needed to control the persistent biliary leak of 200–250 mL/day. Liver function was evaluated again. The indocyanine green retention rate at 15 min was 27.7%. LHL15 was 0.575. Liver volume of segments V and VIII was 260 mL, and remnant liver volume was 1272 mL, which were calculated using the 3D image analysis system (SYNAPSE VINCENT; Fuji Photo Film Co., Ltd.). Functional remnant liver volume (FRLV) was calculated based on the liver volume using gadolinium-ethoxybenzyl-diethylenetriamine pentaacetic acid-enhanced MRI for 20 min [11]. FRLV after liver resection of segments V and VIII was 2176 mL. Even if the function becomes extinct by PTPE to segment V and VIII, remnant liver volume was sufficient. We considered that bile leakage could not be cured with either PTPE or bile duct ablation alone, because of the large volume of bile leakage more than 200 mL per day. The therapeutic strategy involved combination therapy of PTPE and bile duct ablation. First, percutaneous transhepatic cholangiography drainage (PTCD) tube was inserted to the bile duct of segment V. PTPE with coil embolization was performed to the part of the portal vein of segments V and VIII by puncturing the part of the portal vein of segment V. The liver volume of segments V and VIII was decreased from 260 to 123 mL after PTPE. After the PTPE, bile leakage decreased to about 50 mL/day. We confirmed that cholangiography via the PTCD tube showed the bile duct at segments V and VIII. For bile duct ablation, 1.2 mL pure ethanol was injected from the PTCD tube. Over 1.2 mL of ethanol leaked into the abdominal cavity. After ethanol injection, the PTCD tubes were clamped for 5 min. Then, another bile duct ablation with 2.0 mL of pure ethanol was performed 1 week after of the first procedure. After bile duct ablation, bile leakage has decreased from 50 to 10 mL/day gradually. The patient left the hospital, and he was rehospitalized and reinjected with 2.0 mL of pure ethanol for three times. The drainage tube and bile duct tube were removed on POD 139 (Fig. 3). The clinical course is summarized in figure. Complications with combination treatment of PTPE and bile duct ablation were not noted.

## Discussion

Bile leakage is categorized into four types by Nagano et al.: type 1 is minor leakage from the cut surface, type 2 is leakage caused by insufficient closure of the bile duct stump, type 3 is leakage from the injured bile duct wall at exposed bile duct or hilar bile duct, and type 4 is leakage from the distal orifice of the isolated bile duct [5, 9]. The present case was classified as type 4.

Therapeutic methods for isolated bile leakage can be divided into surgical and non-surgical methods. Surgical treatment was adapted to intractable bile leakage which was not treated by non-surgical management. Fukuhisa et al. reported the successful treatment of a case by surgical treatment of the isolated bile leakage from the Spiegel lobe after right hemihepatectomy [12]. They suggested that surgical treatment with liver resection for postoperative isolated bile leakage was both a quick and reliable procedure for patients with acceptable liver function and anatomical subject [12]. Fistulojejunostomy was one of the therapeutic methods reported [13], but a detailed report is unavailable. In this patient, isolated bile leakage was diagnosed using DIC-CT and cholangiography, with the drainage tube or endoscopic method. It was considered that it was not cured by drainage alone. Anterior resection after partial hepatectomy was not technically easy, and the patient was not hoped. Fistulojejunostomy to the leaked bile duct was difficult because the sutured space was transformed into a slit shape. Furthermore, this operation was thought to be difficult for a patient with hepatocellular carcinoma and cirrhosis because treatments such as transcatheter arterial chemoembolization or radiofrequency ablation for the recurrence of HCC are contraindication because treatment with transarterial chemoembolization and radiofrequent ablation becomes high risk of the liver abscess [14].

Non-surgical treatment for isolated bile leakage requires interventions such as PTPE, embolization with fibrin glue, and bile duct ablation. PTPE was one of the therapeutic methods for intractable bile leakage, and the use of PTPE decreased bile leakage quantity due to the embolus of the responsible bile duct domain. Sadakari et al. reported a successfully treated case of bile leakage from isolated bile duct of the posterior segment by PTPE which induces atrophy of hepatocytes [15]. Tanaka et al. reported that the isolated bile leakage was cured by sealing the duct with a mixture of fibrin glue and iodized oil [16]. However, this treatment did not cause infection, but a few bile leakages were noted. Bile duct ablation therapy was reported for the first time by Kyokane et al. [17].

When segments V and VIII were resected, the FRLV was 2176 mL. Itoh et al. showed the cutoff value of liver-related morbidity was 731 mL [11]. Therefore, it was thought that it has a low risk of liver failure even if segments V and VIII of the liver became dysfunctional. Previous reports have shown successful treatment of bile leakage with bile duct ablation when the amount of bile leakage was 50–150 mL [18–20]. PTPE was performed when the amount of bile leakage was 40–50 mL [15, 21]. In this case, the amount of bile leakage from segments V and VIII was more than 200 mL/day. It was considered that PTPE or bile duct ablation therapy alone could not stop the bile leakage. PTPE reduces hepatocellular function and decreases bile production. Kyokane reported that, in an animal study, selective intrahepatic biliary ethanol injection destroyed the biliary epithelium, permeated the parenchyma, induced hepatocyte degeneration, and resulted in compensatory hypertrophy of the non-injective hepatic lobe [22]. We thought that it was appropriate to perform bile duct ablation after decreasing bile production with PTPE. We performed the combination therapy with PTPE and bile duct ablation with pure ethanol. First, a PTCD tube was inserted into the leaking bile duct for a direct route, applying pure ethanol, which had originally been planned after PTPE. Because we expected difficulties in inserting the PTCD tube into the atrophic liver after PTPE, the PTCD tube was placed before PTPE. Previous reports showed that inserting the PTCD tube into the leaking bile duct was difficult [20, 21]. To the best of our knowledge, no report was found of successful PTCD tube insertion into the atrophic liver after PTPE. The insertion of the PTCD tube into the bile duct of segment V was easier than the insertion into the bile duct of segment VIII, because the liver shape changed after hepatectomy and the bile duct of segment VIII was near the cavity of the bile leakage, making insertion technically difficult. In the past report, the retrograde transhepatic biliary drainage tube was inserted into the bile duct under open surgery, and bile duct ablation with ethanol was performed from the tube [20]. In this case, 6-Fr PTCD tube was successfully inserted to the normal size of the bile duct of segment V by an interventional radiologist before PTPE. PTPE decreased the liver capacity of segments V and VIII. Bile leakage was decreased from 150 to 50 mL. Bile leakage was not cured by PTPE alone. Although bile duct ablation was performed with pure ethanol, this case report revealed that bile leakage was treated with PTPE because it was not cured by bile duct ablation alone [15].

TAE for treating bile leakage has been rarely reported, and the authors found only one case report [23]. However, it was reported that TAE can increase the risk of biliary infection, which may cause liver abscess after the procedure [14]. Therefore, TAE was not performed in our case.

## Conclusions

We report a case of isolated bile leakage after partial hepatectomy that was successfully treated with combination therapy with PTPE and bile duct ablation with ethanol. It is important to diagnose the bile leakage type and to select the adequate treatment method.

## Abbreviations

CT: Computed tomography
DIC: Drip infusion cholangiography
FRLV: Functional remnant liver volume
MRI: Magnetic resonance imaging
POD: Postoperative day
PTCD: Percutaneous transhepatic cholangiography drainage
PTPE: Percutaneous transhepatic portal embolization
